# Cholesterol Synthetase DHCR24 Induced by Insulin Aggravates Cancer Invasion and Progesterone Resistance in Endometrial Carcinoma

**DOI:** 10.1038/srep41404

**Published:** 2017-01-23

**Authors:** Miao Dai, Xiao-Lu Zhu, Fei Liu, Qin-Yang Xu, Qiu-Lin Ge, Shu-Heng Jiang, Xiao-Mei Yang, Jun Li, Ya-Hui Wang, Qing-Kai Wu, Zhi-Hong Ai, Yin-Cheng Teng, Zhi-Gang Zhang

**Affiliations:** 1 Department of Obstetrics and Gynecology, Shanghai Jiao Tong University Affiliated Sixth People’s Hospital, No. 600 Yishan Road, Shanghai 200233, P. R. China; 2 State Key Laboratory of Oncogenes and Related Genes, Shanghai Cancer Institute, Ren Ji Hospital, School of Medicine, Shanghai Jiao Tong University, Shanghai 200240, P. R. China

## Abstract

3β-Hydroxysteroid-Δ24 reductase (DHCR24), the final enzyme of the cholesterol biosynthetic pathway, has been associated with urogenital neoplasms. However, the function of DHCR24 in endometrial cancer (EC) remains largely elusive. Here, we analyzed the expression profile of DHCR24 and the progesterone receptor (PGR) in our tissue microarray of EC (n = 258), the existing EC database in GEO (Gene Expression Omnibus), and TCGA (The Cancer Genome Atlas). We found that DHCR24 was significantly elevated in patients with EC, and that the up-regulation of DHCR24 was associated with advanced clinical stage, histological grading, vascular invasion, lymphatic metastasis, and reduced overall survival. In addition, DHCR24 expression could be induced by insulin though STAT3, which directly binds to the promoter elements of DHCR24, as demonstrated by ChIP-PCR and luciferase assays. Furthermore, genetically silencing DHCR24 inhibited the metastatic ability of endometrial cancer cells and up-regulated PGR expression, which made cells more sensitive to progestin. Taken together, we have demonstrated for the first time the crucial role of the insulin/STAT3/DHCR24/PGR axis in the progression of EC by modulating the metastasis and progesterone response, which could serve as potential therapeutic targets for the treatment of EC with progesterone receptor loss.

Endometrial cancer (EC) is the most common gynecologic malignancy of the female reproductive tract in developed countries^1^. More than 47,000 women are diagnosed with endometrial carcinoma in the United States each year^2^,^3^, Numerous epidemiological studies have confirmed that metabolic factors are closely associated with various cancers, particularly EC^4^,^5^,^6^. Hyperinsulinemia has been considered as an EC risk factor independent of estradiol^7^, and metformin, an insulin-sensitizer, is found to diminish EC proliferation and has positive effects on cancer clinical evolution^8^,^9^. The majority of EC cases diagnosed in the early stage have a favorable prognosis. However, women diagnosed with advanced stage, poor differentiation, and progesterone receptor negative EC have a higher risk of metastasis and a poor prognosis^3^. Hormonal therapy, such as medroxyprogesterone acetate (MPA), has been applied in the conservative treatment of young patients who wish to preserve their fertility, as well as in the palliative treatment of advanced-state patients^10^. However, the administration of progesterone to EC patients does not work effectively because of de novo or acquired progestin resistance. To date, more than 30% of EC patients with progestin treatment have presented progestin resistance^11^. Over the past decades, little improvement has been demonstrated in this area. Thus, it is imperative to identify novel, effective anticancer targets with prognostic value to accurately predict the survival of EC patients and to cure this disease efficiently.

3β-Hydroxysteroid-Δ24 reductase (DHCR24), the final enzyme of the cholesterol biosynthetic pathway, catalyzes the reduction of the Δ24 double bond in desmosterol to produce cholesterol^12^. DHCR24 is involved in multiple cellular functions, such as oxidative stress, cell differentiation, anti-apoptotic function, and anti-inflammatory activity^13^. In addition, DHCR24 is dysregulated in various tumors including prostate cancer, ovarian cancer, and urothelial carcinoma^14^,^15^,^16^. The up-regulation of DHCR24 is associated with invasiveness and disease recurrence in urothelial cancer, which suggests a crucial role for this protein in tumor progression^14^. Although DHCR24 has been found to take part in tumor progression, the expression pattern of DHCR24 in EC is still not fully elucidated. Moreover, the regulatory mechanisms by which DHCR24 mediates its functions as well as its involvement in progestin resistance are unclear.

A new era of EC research has begun as clinically relevant genomic information has been gradually elucidated^17^,^18^. Herein, we analyzed the expression pattern of DHCR24 at both the mRNA and protein levels in a large set of clinical samples of EC combined with TCGA (the Cancer Genome Atlas, which generates comprehensive, multidimensional maps of the key genomic changes in 33 types of cancer) and GEO datasets (Gene Expression Omnibus, which is a public functional genomics data repository)^19^. We explored the relationship between DHCR24 expression and the corresponding clinical parameters. We demonstrated that DHCR24 overexpression exhibited characteristics of metastasis and progestin resistance. Furthermore, we found for the first time that insulin, the peptide hormone produced by beta cells of the pancreatic islets, could enhance the transcription of cholesterol synthetase DHCR24 via STAT3 in EC. These experimental data in support of the insulin/STAT3/DHCR24/PGR axis reveal characteristics of an important pathway involved in both metastasis and progesterone response in EC.

## Results

### DHCR24 is up-regulated in EC

**Figure 1:**
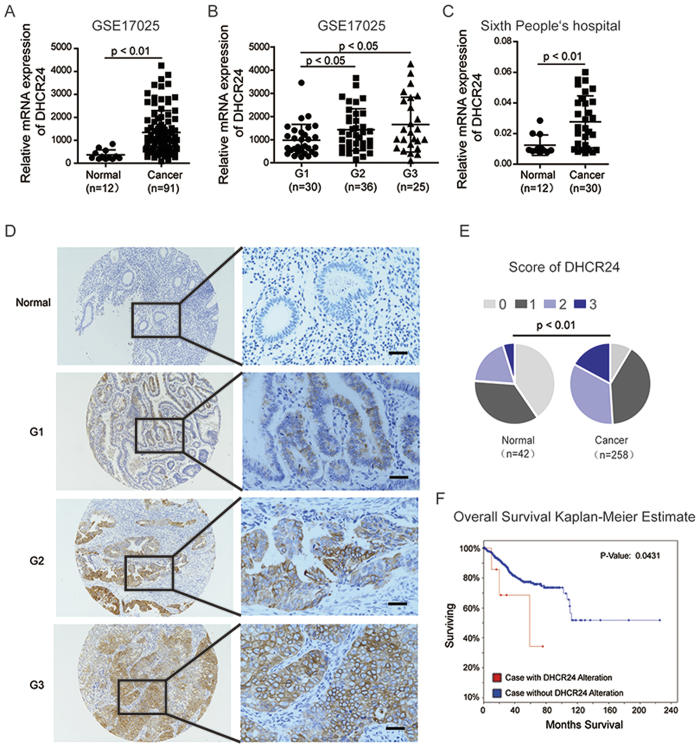
DHCR24 is up-regulated in endometrial cancer. (**A**) mRNA expression level of DHCR24 in 91 EC tissues and 12 non-tumor tissues in the GEO database. (**B**) Differential mRNA expression level of DHCR24 in the histological grading of the GEO database. n, number of analyzed samples. (**C**) mRNA expression level of DHC24 in freshly frozen endometrial cancer tissues. (**D**) Representative IHC staining of DHCR24 in the tissue microarray (TMA) of endometrial cancer from histological Grade 1 to Grade 3 of EC specimens. Scale bar, 50 μm. (**E**) Score of the IHC stained in the TMA of EC. Distribution of DHCR24 in the cytoplasm was quantified in EC. Scoring was conducted based upon the percentage of positively stained cells: 0–10% scored 0; 10–35% scored 1; 36–70% scored 2; and more than 70% scored 3. (**F**) Overall survival Kaplan-Meier estimate of DHCR24 alterations in TCGA (DHCR24 alteration group: Red line, No DHCR24 alteration group: Blue line).

We first analyzed DHCR24 expression in the GEO database of 91 endometrial cancer and 12 normal tissues^19^. We observed that DHCR24 was significantly over-expressed in EC tissues compared with their normal counterparts (Fig. 1A). Importantly, we found that DHCR24 expression increased in tissues from different grades of EC (Grade 1, Grade 2, and Grade 3) (Fig. 1B). Consistent with the data from the GEO database, DHCR24 expression was also up-regulated in the EC patients of the cohort from our hospital at the mRNA level in EC patients of the cohort from our hospital (Fig. 1C). Subsequently we detected the subcellular localization of DHCR24 by immunohistochemistry (IHC) in two tissue microarrays of 258 EC samples and 42 noncancerous samples. Expectedly, the protein levels of DHCR24 in the tissues of the EC patients were also significantly higher than those of the normal controls (Fig. 1D,E). Furthermore, the Kaplan Meier analysis of the patients’ follow-ups in the corpus endometrioid carcinomas Dataset (n = 548) from TCGA showed that the overall survival in EC patients with the DHCR24 gene alteration was significantly shorter than those without the DHCR24 alteration (*p* < 0.05) (Fig. 1F).

### High DHCR24 expression predicts poor prognosis in EC

We next investigated the relationship between the protein levels of DHCR24 and patient clinical parameters in EC (Table 1). High expression of DHCR24 in the EC was associated with histological grade (*p* < 0.001), FIGO (International Federation of Gynecology and Obstetrics) stage (*p* < 0.001), vascular invasion (*p* = 0.025), and LN metastasis (*p* = 0.006). However, no significant correlation was observed between DHCR24 levels and age, pregnancy, family history, and pathological type (Table 1, *p* > 0.05).

**Figure 2:**
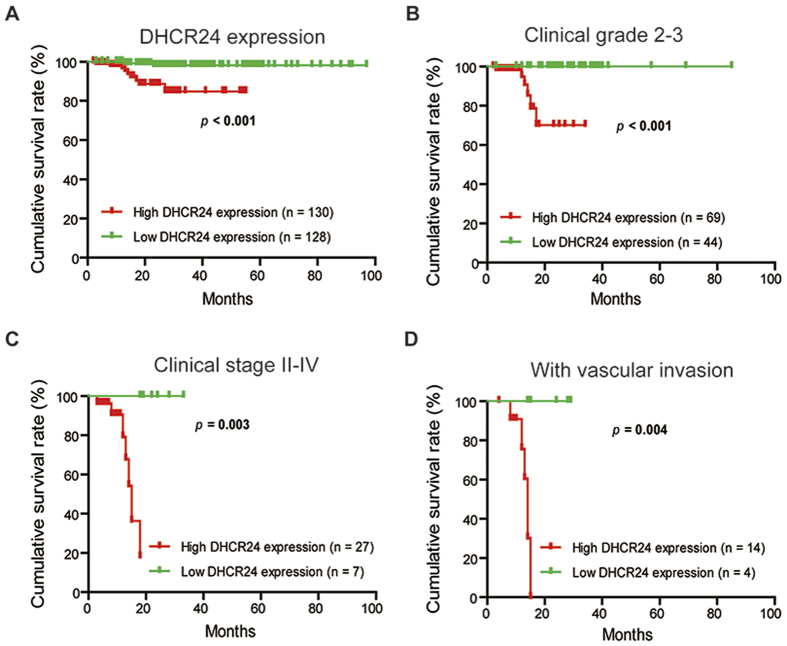
High DHCR24 expression predicts poor prognosis in the two tissue microarrays of EC. (**A**) Comparisons of the overall survival (OS) between DHCR24 low- and high-expression groups in EC. (**B**) Comparisons of the OS between DHCR24 low- and high-expression groups in the histological Grade 2 and Grade 3 cohort. (**C**) Comparisons of the OS between DHCR24 low- and high-expression groups in the clinical stage II-IV cohort. (**D**) Comparisons of the OS between DHCR24 the low- and high-expression groups in the vascular invasion cohort.

Based on its association with aggressive tumor behaviors, DHCR24 may serve as a novel potential prognostic marker. To determine the prognostic value of DHCR24 in EC, the correlation between the expression of DHCR24 and clinical follow-up information was assessed by a Kaplan-Meier analysis and a log-rank test. As shown in Fig. 2A, high DHCR24 expression was associated with poor overall survival (*p* < 0.001). We further analyzed the relationship between DHCR24 expression and the overall survival in EC patients with the advanced EC who classified as Grades 2 and 3 or in stages II, III, and IV. These results showed that the overall survival was much shorter in the advanced EC patients with high DHCR24 expression than those with low DHCR24 expression (Fig. 2B,C). Similar results were also obtained in EC patients with vascular invasion (Fig. 2D).

### DHCR24 could be induced by insulin stimulation via STAT3 in EC

**Figure 3:**
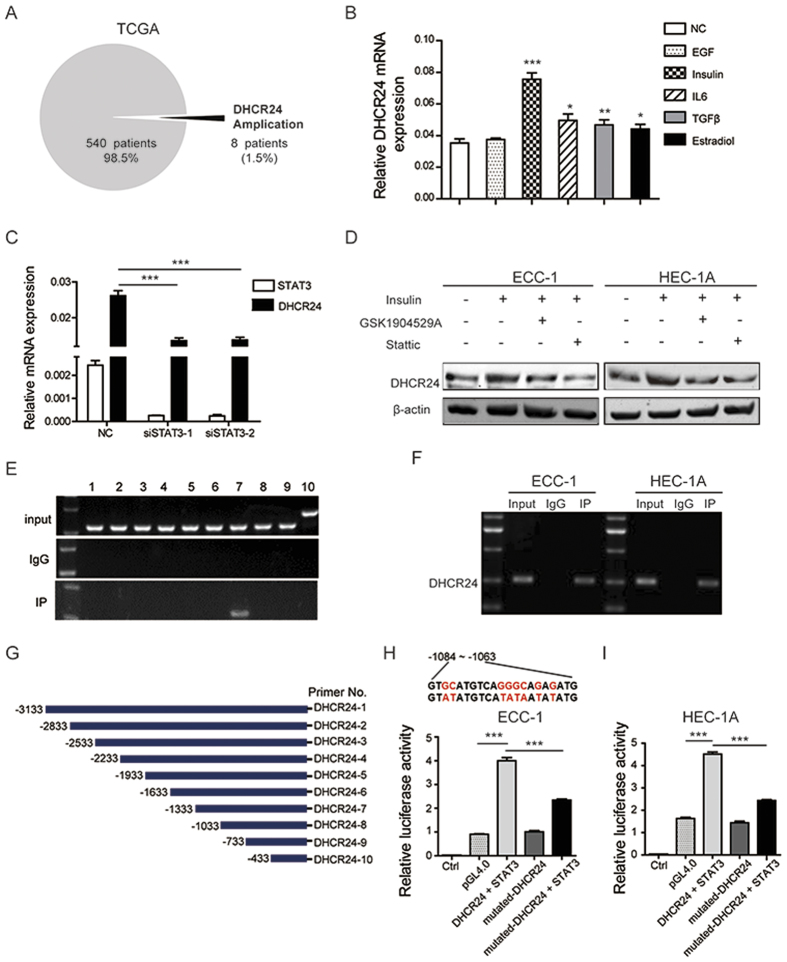
DHCR24 could be induced by insulin stimulation via STAT3 in EC. (**A**) DHCR24 DNA copy number amplification from the genomic-scale sequencing of EC in TCGA. (**B**) mRNA expression of DHCR24 stimulated by EGF, insulin, IL6, TGF-β and estradiol evaluated by qPCR. (**C**) The mRNA expression level of DHCR24 and STAT3 under STAT3 siRNA interference. (**D**) The protein level of DHCR24 was determined in ECC-1 and HEC-1A cells treated with insulin, GSK1904529A and Stattic by immunoblotting (cropped images are shown here, the non-cropped images are shown in Supplementary data 6). (**E**) A ChIP assay was performed to confirm the potential STAT3 binding site in the DHCR24 promoter region. IgG and input fraction were used as controls. (**F**) A ChIP assay was performed in two endometrial cancer cell lines, HEC-1A and ECC-1. (**G**) In total, 10 pairs of primers were constructed according to the promoter of DHCR24. (**H** and **I**) A Luciferase reporter assay was performed using ECC-1 and HEC-1A cells after transfecting the wild type plasmids and mutated plasmids (mutation site: *red*). The data shown are the mean ± SD. (*p < 0.05; **p < 0.01; ***p < 0.001).

DNA copy number gains play an important role in gene overexpression. In an attempt to identify whether gene amplification contributes to the higher expression of DHCR24 in EC, we analyzed the DHCR24 alterations in the genomic-scale sequencing of EC in TCGA. We found that DHCR24 gene amplification was identified in only 8 of 548 samples (1.5%) (Fig. 3A), insufficient evidence to explain the overexpression of DHCR24. It has been reported that tumor microenvironment factors, such as cytokines and growth factors, are involved in cancer cell metastasis and the response to progestin in EC^20^,^21^. EGF, insulin, IL6, TGF-β, and estradiol were applied to stimulate EC cells to investigate whether these factors contribute to the overexpression of DHCR24. Our results suggest that insulin can significantly induce the expression of DHCR24 (Fig. 3B). This coincides with the fact that chronic hyperinsulinemia from insulin resistance is involved in endometrial tumorigenesis. Therefore, the alteration of DHCR24 expression in EC could be caused by the tumor microenvironment.

To identify which transcription factor was responsible for the insulin induced overexpression of DHCR24, we searched for downstream transcription factors of insulin that are also known to be involved in the regulation of cell metastasis and the response to progestin. Previous studies have shown that STAT3 is found in hepatocytes and multipotent progenitors^22^,^23^,^24^. Aiming to further examine whether STAT3 participates in the up-regulation of DHCR24 expression induced by insulin in EC cells, we first examined the DHCR24 expression after STAT3 silencing. Our data showed that STAT3 knockdown could decrease the expression of DHCR24 (Fig. 3C). In addition, the insulin receptor (IR) inhibitor GSK1904529A and STAT3 activity inhibitor Stattic could block the increased level of DHCR24 induced by insulin (Fig. 3D). To demonstrate whether STAT3 could directly regulate the expression of DHCR24, we constructed 10 pairs of primers according to the promoter of DHCR24, and we performed ChIP PCR in ECC-1 cells to evaluate whether STAT3 could directly bind to the promoter of DHCR24 (Fig. 3G). As expected, ChIP analysis indicated that STAT3 directly bound to 7 regions in the DHCR24 promoter (Fig. 3E). The same results were found in HEC-1A and ECC-1 cells (Fig. 3F). Reporter assays further confirmed that the transcriptional activity of the DHCR24 promoter was significantly induced by STAT3 (*p* < 0.001), and was significantly decreased by a DHCR24 promoter mutation (Figs. 3H,I). These data suggest that STAT3 can directly regulate the expression of DHCR24 at the transcriptional level in EC cells.

### A high expression level of DHCR24 promotes cell invasion and migration in EC cells

**Figure 4:**
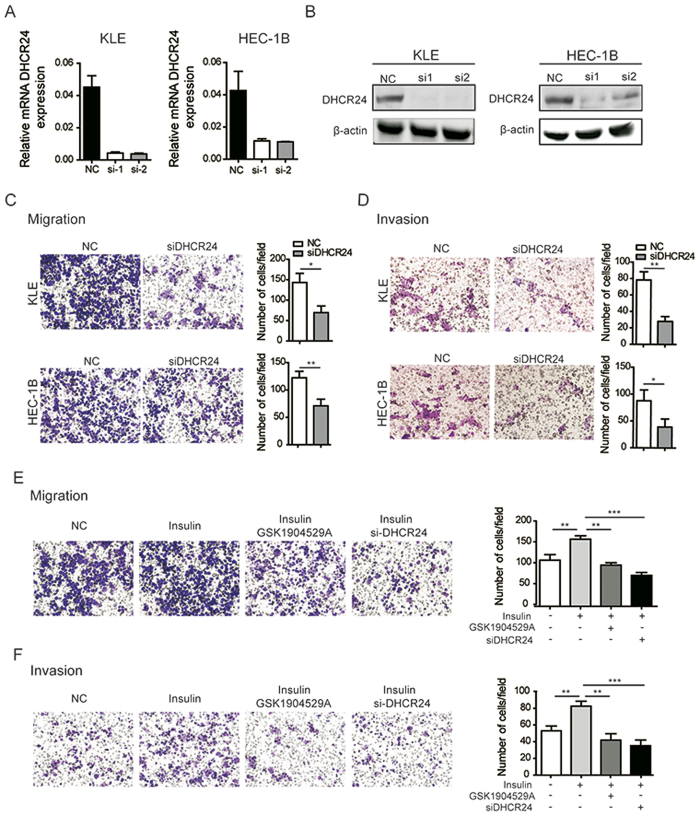
Silencing of DHCR24 suppresses EC cell invasion and migration. (**A**) qRT-PCR analysis of the DHCR24 expression after DHCR24 siRNA interference. (**B**) Successful DHCR24 silencing was confirmed by immunoblotting. (**C**) The level of migration was probed in the DHCR24-silenced group and the DHCR24 control group derived from KLE and HEC-1B cells. (**D**) The level of invasion was assessed in the DHCR24-silenced group and the DHCR24 control group derived from KLE and HEC-1B cells. (**E**) The level of migration ability of HEC-1B cells treated with insulin, GSK1904529A, and the silencing of DHCR24 was determined using a transwell assay. (**F**) The invasion ability of HEC-1B cells treated with insulin, GSK1904529A, and silencing of DHCR24 was probed. The data are presented as the mean ± SD of three independent experiments. (*p < 0.05; **p < 0.01; ***p < 0.001).

A significant correlation was observed between DHCR24 overexpression and aggressive tumor behaviors in EC, which suggests that DHCR24 may play a pivotal role in EC tumor development. To test this hypothesis, siRNA-mediated loss-of-function for DHCR24 was performed in EC cells. DHCR24-depleted transfectants showed significantly decreased expression levels of DHCR24 compared to control cells by RT-PCR analysis and Western blot (Fig. 4A,B, respectively). Subsequently, we detected the migration and invasion of KLE cells and HEC-1B cells after the silencing of DHCR24. The results showed that the migration and invasion of EC cells were significantly decreased after the silencing of DHCR24, indicating that DHCR24 may play an important role in EC metastasis (Fig. 4C,D). Consistently, the treatment of EC cells with insulin resulted in the increased migration and invasion ability. The enhanced activity induced by insulin could be blocked by the insulin receptor (IR) inhibitor GSK1904529A and the silencing of DHCR24 (Fig. 4E,F).

### DHCR24 expression has a negative correlation with PGR expression

**Figure 5:**
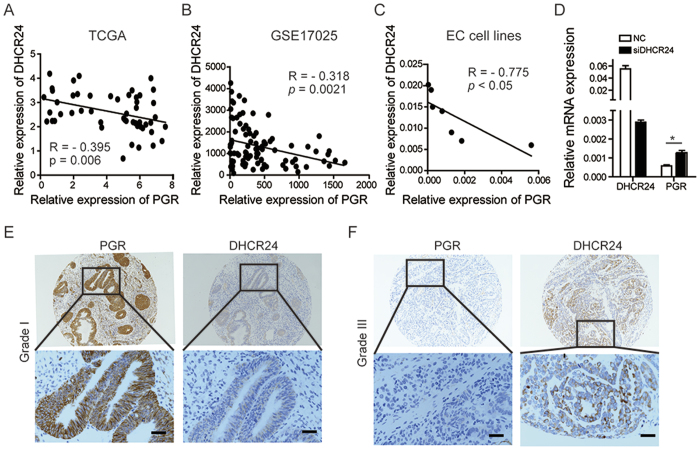
DHCR24 expression level is negatively correlated with PGR expression level. (**A**) The correlation between the expression level of DHCR24 and PGR was analyzed in the TCGA dataset of EC (R = −0.395, *p* = 0.006). (**B**) The correlation between the expression levels of DHCR24 and PGR was analyzed in the GEO dataset of EC (R = −0.318, *p* = 0.0021). (**C**) The correlation between the expression levels of DHCR24 and PGR was analyzed in the EC cell lines (R = −0.775, *p* < 0.05). (**D**) mRNA expression level of DHCR24 and PGR under DHCR24 siRNA interference. (**E**) Representative images of the immunohistochemical staining for DHCR24 and PGR in paraffin continuous sections of Grade I endometrial cancer. (**F**) Representative images of immunohistochemical staining for DHCR24 and PGR in paraffin continuous sections in Grade III endometrial cancer. Scale bar 50 μm.

As DHCR24, the final enzyme of the cholesterol biosynthetic pathway, is closely related with hormone production, we wondered whether DHCR24 correlated with the progesterone receptor. Interestingly, a significantly negative correlation was found between the expression levels of DHCR24 and the progesterone receptor by analyzing the GEO datasets and TCGA datasets (*p* = 0.006, *p* = 0.0021, respectively) (Fig. 5A,B). Similar results were also found in EC cell lines (Fig. 5C). Then, we noticed that after DHCR24 silencing with siRNA interference, PGR expression was significantly up-regulated (*p* < 0.05) (Fig. 5D). Furthermore, we performed IHC analysis with paraffin continuous tissue sections. Samples of Grade I EC had strong staining intensity for PGR, whereas there was barely any DHCR24 staining (Fig. 5E). Positivity staining of DHCR24 was observed in Grade III EC, but PGR staining was weak (Fig. 5F). Together, these data indicated that DHCR24 expression has a negative correlation with PGR expression at both the mRNA and protein levels.

### DHCR24 silencing enhances the sensitivity of EC cells to medroxyprogesterone acetate treatment

**Figure 6:**
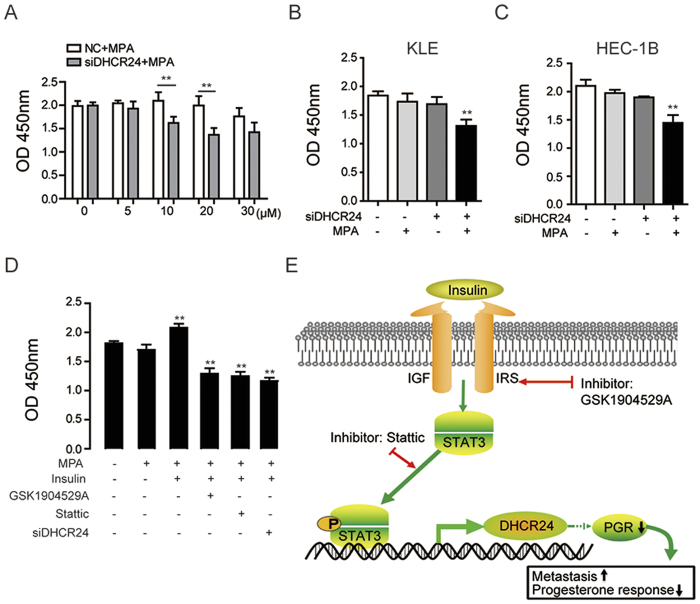
Silencing of DHCR24 enhances the sensitivity of endometrial cancer cells to medroxyprogesterone acetate treatment. (**A**) The cell viability of HEC-1B cells was determined in the DHCR24-silenced group and the control group treated with different concentration of MPA ranging from 0 to 30 μM. (**B**) The cell viability of DHCR24-silenced and control KLE cells was measured after treatment with MPA (10 μM). (**C**) The cell viability of DHCR24-silenced and control HEC1B cells was measured after treatment with MPA (10 μM). (**D**) The cell viability was determined in HEC-1B cells treated with MPA, insulin, GSK1904529A or Stattic or with silenced DHCR24. (**E**) Schematic summary of the findings presented in this study on the role of DHCR24 in endometrial cancer. Insulin up-regulated DHCR24 expression via STAT3, directly binding to the DHCR24 promoter region, which modulated cell metastasis and the response to progestin therapy. (**p < 0.01).

As DHCR24 expression was negatively correlated with PGR expression, we speculated that DHCR24 may affect the response of EC cells to progestin. To probe this possibility, DHCR24-silenced and control EC cells were treated with different concentrations of MPA, and 10 μM was used for further experiments (Fig. 6A). The data showed that the cell viability of DHCR24-silenced EC cells (KLE and HEC-1B) was significantly decreased compared to the control (Fig. 6B,C), indicating that DHCR24 silencing improved the progesterone responsiveness of the cells. On the contrary, insulin treatment reduced the response to MPA (Fig. 6D). The effect of insulin on progesterone responsiveness could be reversed by GSK1904529A (an inhibitor of the insulin receptor), Stattic (an inhibitor of STAT3), and the silencing of DHCR24 (Fig. 6D). Taken together, these data supported that the insulin/STAT3/DHCR24 axis plays an important role in the metastasis and response to progestin of EC (Fig. 6E).

## Discussion

Previous studies have shown that DHCR24 expression is deregulated in different types of cancer. The up-regulation of DHCR24 has been observed in prostate, hepatocellular carcinoma, and metastatic melanoma^16^,^25^,^26^. Conversely, DHCR24 was found to be down-regulated in adrenal cancer^27^. Our study showed that DHCR24 was highly expressed in EC tissues compared with normal tissue at both the mRNA and protein levels, which was in line with the data found in the GEO dataset of EC. In addition, higher DHCR24 expression was observed in the patients with a higher histology grade, and indicated a poor prognosis. As noted above, DHCR24 alteration is related to tumor progression and may serve as a novel prognostic factor and potential biomarker for endometrial cancer.

Elevated expression DHCR24 was observed in different neoplastic diseases. Androgen could mediate the expression of DHCR24 by binding to its promoter in prostate cancer^26^. The reason why DHCR24 is up-regulated in EC is unclear. Various factors are involved in the regulation of gene expression. We excluded the possibility that DNA copy number amplification contributed to the elevated expression of DHCR24 because of the low proportion of DHCR24 amplification in EC. It is widely believed that the tumor microenvironment is involved in tumor progression^28^, which promoted us to explore the possibility that extracellular signaling regulates DHCR24 expression. In this study, we found that an important factor, insulin, could induce the overexpression of DHCR24 in endometrial cancer cells, which agrees with the finding that hyperinsulinemia is quite prevalent in EC^7^. Accordingly, insulin could significantly increase cell metastasis, and these effects were reversed by the silencing of DHCR24. Several studies have reported that STAT3 is necessary for cell development, metastasis, and prognosis^29^,^30^. Our data suggested that STAT3 was required for insulin-induced DHCR24 expression. STAT3 knockdown could inhibit the increased levels of DHCR24, and the STAT3 inhibitor Stattic could suppress the level of DHCR24 induced by insulin, indicating that STAT3 may be hijacked by insulin signaling to regulate DHCR24 expression. Accordingly, we found that there are a series of STAT3 binding sites in the promoter region of DHCR24. We confirmed that STAT3 could bind to the promoter region of DHCR24 and promote its transcription in EC cells, using ChIP analysis and a promoter reporter assay. Overall, these data suggest that insulin is an important extracellular signal that could modulate DHCR24 expression via STAT3 to promote cell metastasis and progestin resistance in EC. Hyperinsulinemia in insulin resistance is one of the proposed mechanisms that explains the obesity-cancer link and has a detrimental effect on survival in cancer^7^,^31^,^32^. Understanding the insulin signaling pathway involved in cell metastasis and progestin resistance will be critical for the development of new intervention strategies to prevent or treat EC associated with obesity.

DHCR24 is a multi-functional protein involved in various biological processes. Hence, we explored the biological behaviors of DHCR24 in EC cells. Our functional studies showed the involvement of DHCR24 in cell invasion and migration, which was in line with previous reports showing its role in metastasis^33^. Furthermore, consistent with the GEO and TCGA datasets, DHCR24 expression negatively correlated with PGR expression in our TMA of EC. To further investigate the link between DHCR24 and the response to progestin, we performed functional studies in EC cells following the administration of medroxyprogesterone acetate. We observed that DHCR24 silencing could significantly up-regulate the expression of PGR, which cause EC cells to be more sensitive to MPA treatment, suggesting that DHCR24 could be targeted to develop pharmacological strategies to enforce responsiveness to progestin in EC patients with progestin resistance.

Interestedly, the expression of PGR is negatively correlated with the expression of DHCR24 in this study. DHCR24 is the final enzyme of cholesterol synthesis, and cholesterol is the essential precursor for all steroid hormones, include progesterone^13^,^34^. It has been reported that constant progestin stimulation gave rise to a resistance effect to progestin and reduced the expression of PGR^35^. Therefore, we speculate that DHCR24 might display negative feedback on the PGR. Although we have found the inverse correlation between PGR and DHCR24 by combination of clinical and experimental data, how the PGR is connected DHCR24 and which molecules are involved in remain elusive. Further studies need to unveil the potential intermediates and underlying mechanism involved in connection between PGR and DHCR24, which will provide a new strategy to solve the problem of progestin resistance and finally improve the treatment of EC patients.

Although insulin could induce DHCR24 expression in EC cells in our study, a lack of correlation in the patient serum insulin level and the body mass index data in our study limits the ability to analyze the clinical relationship between insulin and DHCR24. Future experiments will address these shortcomings.

In summary, our study illustrated that the overexpression of DHCR24 was a prognostic factor for the progression of EC patients, which suggests that DHCR24 could serve as a novel molecular predictor in EC. In addition, we demonstrated for the first time that DHCR24-mediated metastasis and the response to progestin could be controlled by insulin via STAT3, which directly bind to the DHCR24 promoter and activate its transcription. Thus, targeting the insulin/STAT3/DHCR24 axis may provide a novel strategy for the prevention of EC metastasis and the treatment of EC associated with progestin resistance.

## Material and Methods

### Clinical sample and database

Two tissue microarrays (TMA) containing 258 cases of tumor tissues and 42 non-tumor tissues were constructed by ZhuoLi Biotech Co., Ltd. in Shanghai, China. All paraffin-embedded samples with complete follow-up information were obtained from Shanghai Jiao Tong University Affiliated Sixth People’s Hospital in China, and the First People’s Hospital of Huai’an City in Jiangsu, China between April 2003 and March 2013. An additional 30 freshly frozen EC tissues and 12 non-cancerous endometrial tissue samples were collected between April 2008 and March 2014 from Shanghai Jiao Tong University Affiliated Sixth People’s Hospital in China. Non-cancer samples were obtained from patients with non-malignant diseases (uterine leiomyoma, endometrial polyps, and endometrial hyperplasia) that underwent hysterectomy or hysteroscopy. All of them gave written informed consent, and all the experiments were approved by the Research Ethics Committees of Shanghai Jiao Tong University Affiliated Sixth People’s Hospital and in accordance with the Declaration of Helsinki. The gene expression profile results, GSE17025, were deposited by Risinger J H *et al*. (GSE17025, Gene Expression Omnibus database (GEO), http://www.ncbi.nlm.nih.gov/geo/) in the National Center for Biotechnology Information (NCBI)^19^. We subsequently performed analyses on the database from corpus endometrioid carcinomas in the Cancer Genome Atlas (TCGA, http://www.cbioportal.org/).

### Cell culture

Human endometrial cancer cell lines KLE, HEC1-A, RL95–2, ISHIKAWA, HEC1-B, AN3CA, and ECC-1 were all preserved at Shanghai Cancer Institute. All these cells were cultured in specific medium (Invitrogen, Carlsbad, CA, USA) supplemented with 10% (v/v) fetal bovine serum (FBS, Invitrogen), 100 μg/ml streptomycin, and 100U/ml penicillin (Invitrogen) at 37 °C with 5% CO_2_. Cells were serum starved for 12 h prior to EGF (10 ng/ml), insulin (100 nmol/l), TGF-β (5 ng/ml), IL-6 (50 ng/ml), and estradiol (10 nM) treatment for 6 hours^36^,^37^,^38^,^39^. Culture medium was switched to serum-free and phenol red-free medium to avoid potential interference from serum hormones or phenol red. Human recombinant EGF was obtained from R&D (R&D Minneapolis, MN, USA), and insulin, TGF-β, IL-6, and estradiol were obtained from Sigma (Sigma-Aldrich, St. Louis, MO, USA).

### Real-time PCR

The total RNA was extracted from frozen tissue samples or EC cells using TRIzol reagent (Invitrogen) according to the supplier’s protocols. The primers used in this study are shown in Supplementary data 1. Reverse transcription was performed using a Prime-Script RT-PCR Kit (Takara, Tokyo, Japan). The mRNA levels of the detected genes were quantified using an ABI Prism 7500 Sequence Detection System (Applied Biosystems, Inc. USA) with SYBR Green Master Mix (Takara). The 2^−∆Ct^ method was used to quantify the relative expression levels of DHCR24. Experiments were repeated in triplicate independently.

### Western blot

Protein samples were homogenized in a lysis buffer (Beyotime, Suzhou, China) containing proteinase inhibitors and phosphatase inhibitors (Selleck, TX, USA). According to the manufacturer’s protocol, the protein concentration was evaluated using a protein assay reagent kit (Beyotime). Equal amounts of proteins were separated on a sodium dodecyl sulfate polyacrylamide gel and transferred onto polyvinylidene fluoride membranes (Merck Millipore, MA, USA). The membranes were then incubated with the primary antibodies anti-DHCR24 (1:500) (Thermo Fisher Scientific, MA, USA), anti-STAT3 (1: 1,000, Proteintech, IL, USA), anti-β-actin (1: 3,000, Sigma). The proteins were detected using ECL Western Blotting Detection Reagents (Millipore) in accordance with the supplier’s protocol.

### Immunohistochemistry

Tissue samples embedded in paraffin were deparaffinized and rehydrated using xylene and graded ethanol. The sections were blocked with 5% bovine serum albumin for 1 h after antigen retrieval and the neutralization of endogenous peroxidase. The slides were then incubated overnight at 4 °C with the primary antibodies (DHCR24, Thermo Fisher; PGR, Abcam, MA, USA). After washing in phosphate-buffered saline (PBS) for three times, the slide was labeled by a HRP secondary antibody (rabbit) for 1 h at room temperature and again washed three times with PBS. Visualization was performed by DAB and counterstaining with hematoxylin. Scoring was conducted by the percentage of positively stained cells: 0–10% scored 0; 10–35% scored 1; 36–70% scored 2; and more than 70% scored 3. We designated the final score as a high or low expression group as follows: a score of 0 or 1 was considered low expression, and a score of 2 or 3 indicated high expression. The scoring by two senior pathologists was performed in a blind manner.

### Short Interfering RNA–mediated DHCR24 knockdown

Human endometrial carcinoma cell lines KLE and HEC-1-B were maintained in specific medium containing 10% (v/v) FBS and 1% penicillin/streptomycin at 37 °C in humidified atmosphere of 5% CO_2_. Cells were transiently transfected using the Lipofectamine RNAiMAX reagent (Invitrogen), Opti-MEM reduced-serum medium (Invitrogen), and siRNA oligonucleotides (Supplementary data 2). The siRNA (15 nM) treated with 5 μL of RNAiMAX reagent for 48 h was found to be the best concentration with a maximum transfection efficiency (95%) and a maximum silencing of the gene expression of DHCR24. The siRNA-treated cells were used in subsequent experiments after transfection for 48 hrs.

### Cell viability assay

For the drug sensitivity assay, KLE and HEC-1B cells were transfected with DHCR24 siRNA and followed by treatment with MPA at a concentration of 10 μM for 48 hours. Cell viability was measured using a CCK-8 assay (Dojindo, Kumamoto, Japan) according to the manufacturer’s instructions. Cell survival was also measured after treatment with MPA (10 μM), insulin (100 nmol/l), GSK1904529A (60 nM), and Stattic (10 μM). MPA, GSK1904529A and Stattic were obtained from Selleck.

### Migration and invasion assay

Migration and invasion assays were performed as follows: a 200-μL volume of cells at a density of 2 × 10^5^/mL was seeded into the upper chambers (Corning, NY, USA) with an 8-μm pore in 24-well plates. DMEM (Invitrogen) containing 15% FBS as a stimulatory factor was added to the lower chambers. After 12 hours in culture at 37 °C in a 5% CO_2_ atmosphere, the cells were fixed and stained with crystal violet. Then, the cell were counted using a microscope. Five random microscopic fields per well were counted with the double-blind method. For the cell invasion assay, BioCoat Matrigel (BD Biosciences, CA, USA) (300 μg/mL, 100 μL per chamber) was applied to the upper chambers before following the procedure for the migration assay described above.

### ChIP Assay

A Pierce Agarose ChIP Kit was purchased from Thermo Fisher Scientific. Cells were cross-linked with 1% formaldehyde for 10 minutes at room temperature and terminated by adding glycine (1.25 M). Fixed cells were harvested in SDS buffer with a protease inhibitor and then sonicated to generate DNA of 200–1,000 base pairs (bp) in length. The sheared chromatin-lysed extracts were incubated overnight at 4 °C with the anti-STAT3 antibody or control IgG with rotation. After immunoprecipitation (IP) of the cross-linked protein/DNA, the immunocomplexes were reversed to free DNA. PCR was performed with the input DNA or the immunoprecipitates. The PCR products were separated by agarose gel electrophoresis. The primers used for ChIP are listed in the Supplementary data 3.

### Plasmids construction and dual-luciferase reporter assay

DHCR24 promoter-luciferase reporter plasmids containing the DHCR24 promoter region were constructed in the pGL4 plasmid. Wild-type and mutants DHCR24 promoter luciferase constructs were verified by DNA sequencing (Supplementary data 4 and 5). A dual luciferase reporter assay (Promega, WI, USA) was performed according to the manufacturer’s instructions.

### Statistical analysis

The statistical analyses were conducted using the SPSS 19.0 software (SPSS). All experiments were repeated independently at least three times. The results are presented as the means ± SD, and the comparisons were evaluated using a two-tailed paired Student’s t-tests. The relationship between DHCR24 expression and clinicopathological parameters was tested by a Chi-Square and Fisher’s exact tests. For survival analysis, the Kaplan-Meier method was carried out to analyze the correlation between OS and other variables in the GraphPad Prism software (GraphPad). Survival curves were analyzed by the log-rank test. For all tests, p-values < 0.05 were considered to be statistically significant.

## Additional Information

**How to cite this article**: Dai, M. *et al*. Cholesterol Synthetase DHCR24 Induced by Insulin Aggravates Cancer Invasion and Progesterone Resistance in Endometrial Carcinoma. *Sci. Rep.* **7**, 41404; doi: 10.1038/srep41404 (2017).

**Publisher's note:** Springer Nature remains neutral with regard to jurisdictional claims in published maps and institutional affiliations.

## Supplementary Material

Supplementary Information

## Figures and Tables

**Table 1:** Correlation between DHCR24 expression and clinicopathological parameters in 258 patients with endometrial cancer.

	Expression of DHCR24			
	Total	Low (%)	High (%)	P value
**Age**				
<45 years old	45	24(53.3)	21(46.7)	0.625
≥45 years old	213	104(48.8)	109(51.2)	
**Pregnancy**				
Yes	10	4(40.0)	6(60.0)	0.749
No	248	124(50.0)	124(50.0)	
**Family history**				
Yes	11	6(54.5)	5(45.6)	0.767
No	247	122(49.4)	125(50.6)	
**Pathological type**				
Adenocarcinoma	232	120(51.7)	112(48.3)	0.061
squamous carcinoma, papillary serous carcinoma, and clear cell carcinoma	26	8(30.8)	18(69.2)	
**Clinical Stage (FIGO)**				
I	224	121(54.0)	103(46.0)	<0.001*
II	13	5(38.5)	8(61.5)	
III and IV	21	2(9.5)	19(90.5)	
**Grade**				
G1	145	84(54.9)	61(45.1)	<0.001*
G2	83	38(45.8)	45(54.2)	
G3	30	6(20.0)	24(80.0)	
**Vascular invasion**				
Yes	18	4(22.2)	14(77.8)	0.025*
No	240	124(51.7)	116(48.3)	
**LN metastasis**				
Yes	15	2(13.3)	13(86.7)	0.006*
No	243	126(51.9)	117(48.1)	

Abbreviations: LN, Lymph node.

FIGO, International Federation of Gynecology and Obstetrics.

^*^Statistically significant.
