# Supplementary material for: Cholesterol Synthetase DHCR24 Induced by Insulin Aggravates Cancer Invasion and Progesterone Resistance in Endometrial Carcinoma
Source: Sci Rep. 2017 Jan 23;7:41404. doi: 10.1038/srep41404 (PMC5256103; doi:10.1038/srep41404)
Supplement: Supplementary Information [file srep41404-s1.docx]

**Cholesterol Synthetase DHCR24 Induced by Insulin Aggravates Cancer Invasion and Progesterone Resistance in Endometrial Carcinoma**

Miao Dai^1,*^, Xiao-Lu Zhu^1,*^, Fei Liu^1^, Qin-Yang Xu^1^, Qiu-Lin Ge^1^, Shu-Heng Jiang^2^, Xiao-Mei Yang^2^, Jun Li^2^, Ya-Hui Wang^2^, Qing-Kai Wu^1^, Zhi-Hong Ai^1^, Yin-Cheng Teng^1,+^, and Zhi-Gang Zhang^2,+^

^1^Department of Obstetrics and Gynecology, Shanghai Jiao Tong University Affiliated Sixth People’s Hospital, No. 600 Yishan Road, Shanghai 200233, P. R. China. ^2^State Key Laboratory of Oncogenes and Related Genes, Shanghai Cancer Institute, Ren Ji Hospital, School of Medicine, Shanghai Jiao Tong University, 200240 Shanghai, P. R. China.

*These authors contributed equally to this work.

**^+^Correspondence:** Yin-Cheng Teng, Department of Obstetrics and Gynecology, Shanghai Jiao Tong University Affiliated Sixth People’s Hospital

No. 600 Yishan Road, Shanghai 200233, P. R. China, Tel.: +86-21-64369181, E-mail: [teng_yc@126.com](mailto:teng_yc@126.com) and Zhi-Gang Zhang, State Key Laboratory of Oncogenes and Related Genes, Shanghai Cancer Institute, Ren Ji Hospital，School of Medicine, Shanghai Jiao Tong University, No. 800 Dongchuan Road, Shanghai 200240, P. R. China, Tel.: +86-21-34206763; E-mail: [zzhang@shsci.org](mailto:zzhang@shsci.org).

**Supplementary data 1. Primers in this study were as follows:**

| Gene | Primer |
| --- | --- |
| DHCR24 | 5’-GCACAGGCATCGAGTCATCAT -3’ and 5’-GCAGGCTCATCATCAATACGGA-3’ |
| PGR | 5’-CCCAGCATGTCGCCTTAGAAA-3’ and 5’-AGTGCTCTCACAACTCTGACTT-3’ |
| β-actin | 5’-CATGTACGTTGCTATCCAGGC-3’ and 5’-CTCCTTAATGTCACGCACGAT-3’ |

**Supplementary data 2. siRNA oligonucleotides in this study were as follows:**

| Gene | siRNA oligonucleotides |
| --- | --- |
| DHCR24-Homo-495 | 5’- GGAAGUGGACACCAAGAAATT -3’ and 5’- UUUCUUGGUGUCCACUUCCTT -3’ |
| DHCR24-Homo-948 | 5’- CCUGGAUGAGGCUGUCAUUTT -3’ and 5’- AAUGACAGCCUCAUCCAGGTT -3’ |
| DHCR24-Homo-1139 | 5’- GGGAGCUCCAGGACAUUAUTT -3’ and 5’- AUAAUGUCCUGGAGCUCCCTT -3’ |

**Supplementary data 3. Primers used for ChIP to amplify the DHCR24 promoter includes:**

| Gene | Primer |
| --- | --- |
| Set 1 | 5’-tgaagaaaaaaaaaactgtt-3’ and 5’-ttttccagaaggtcatatga-3’ |
| Set 2 | 5’-aaaacaaaactatagtgaca-3’ and 5’-tctgtctccccaaactctaa-3’ |
| Set 3 | 5’-aaagaagaatcagaaatatg-3’ and 5’-gcaacatagcaagaccctgt-3’ |
| Set 4 | 5’-ccaggcttgtcttgaattct-3’ and 5’-tgattattttgggggtgtga-3’ |
| Set 5 | 5’-gaatggttaagcttctaaag-3’ and 5’-tacaaagaaaaaatgcaaaa-3’ |
| Set 6 | 5’-ctaagtctttgaaatccagt-3’ and 5’-cagactgagccttagggcaa-3’ |
| Set 7 | 5’-tgggggtgagagacacataa-3’ and 5’-ggccttgcatacctatccag-3’ |
| Set 8 | 5’-agacagagaagaacctggta-3’ and 5’-tctttttccagttcactgag-3’ |
| Set 9 | 5’-ggaagagtgccagccatagc-3’ and 5’-gcgccccggccctgagtcag-3’ |
| Set 10 | 5’-ctcccagccctccggcggaa-3’ and 5’-ggtgcggcgccgcgcggtaa-3’ |

**Supplementary data 4. Wild-type plasmids of DHCR24 for promoter luciferase**

GTGGCGACTTTCTCTGGCCTAACTGGCCGGTACCTGAGCTCGCTAGCCTCGAGGATATCAAGATCTGGCCTCGGCGGCCAAGCTTTGGGGGTGAGAGACACATAAAGAGACAATTATAAAATAACATGAAAGAACGGTGACAGAGATGCACACTATTTAATGAGCATCTGGATGGGCAGGGGAGTGCTCTAAATGTTTCAGAGGTGGCCCTGAGCTGGCTTCGGAATGTGTGAGGTGAACTCTGAGAAGGGGATGGGAAAGGGCATTCAGAGGTACTAGCTTGACCAGAGGCGCGCAAATGAGGAATGCTGAGGCCCAGGGAGCCTCCTGAGTGCATGTCAGGGCAGAGATGAATGGAGAGGGAACTGGATAGGTATGCAAGGCCAGACAGAGAAGAACCTGGTATGTCATGTTTAGCTTCTAAACTAAAACATCTCGAAAACTTCCTAAAATTCCATATGAAATGCAAATGGCGCTGGATTTCAGCTTTTGCTGGTAACCGTGGCAGCTCCCCTACACTGGTTGCTGAGTGAGGGCAGGGCCATCTGCTTTGCTCACCACCCTACCCGAGGCTTGCAACACTGCCTGGCACAGAGCGGGCCTGGGGAGTTTACTCGGGCTGAAATGCCTTCCTTCATTCTTTCTCCTCCGGGCCTCCCATCTGCCTCAGTGAACTGGAAAAAGAGGAAGAGTGCCAGCCATAGCCTTCCATGCTTCCAATGGCCTCTGGAGTTCACGAACAGGTCTTCACCTTTCTGGGCTGCTTCCAAGTGTACACAATGGAGCTCACCACTCCCCACTGCCCAGGACTGCTGTGTAGCTCAAATGAATTCGTGGAAAGTGCCCCCTAAAAATGAAAAGATGCCTCTGGAGACCTTGCGCACACCGAGTGGCACGACCTGCAGAGGTTACCGCCAGGTTTCCATCCTCCGCGCCCGGGAACCGCCTGTCCGGCCAAAGGGTGACTGACTCAGGGCCGGGGCGCCTCCCAGCCCTCCGGCGGAAAGCCACTGCAAAACCGCTTAAGGCGCGTTTGGAAGAGGCGGCCCCGCAGCCCCGAGGGCCTGGGGCCCTGAGTCCCAACTGCCCACCCCCAGTATTCGCAGCGGACCCCACCGGCACCCAGTCCCTGGCACCCCCGCCTCGCGCGGCGGCGGGGAGAAAAGGGTGGAGCGGTCCGACTCCCGCAGCCAATGAAAGCTGCGGGTTCCTGGTCCGATCCCCGGCGCGGCTCGCCATTGGTCGCCGCCCGGGTCTCGGCCCACCGAACCTCGGCGACCCGAGCCAATCGCGAGGCGGCGGGCGATCCCGGGCTCCCCGGGCTGTGGGCTACAGGCGCAGAGCGGGCCAGGCGCGGAGCTGGCGGCAGTGACAGGAGGCGCGAACCCGCAGCGCTTACCGCGCGGCGCCGCACCAAGCTTGGCAATCCGGTACTGTTGGTAAAGCCACCATGGAAGATGCCAAAAACATAAGAAGCAGCGCC

**Supplementary data 5. Mutant plasmids of DHCR24 for promoter luciferase**

TGGGGGTGAGAGACACATAAAGAGACAATTATAAAATAACATGAAAGAACGGTGACAGAGATGCACACTATTTAATGAGCATCTGGATGGGCAGGGGAGTGCTCTAAATGTTTCAGAGGTGGCCCTGAGCTGGCTTCGGAATGTGTGAGGTGAACTCTGAGAAGGGGATGGGAAAGGGCATTCAGAGGTACTAGCTTGACCAGAGGCGCGCAAATGAGGAATGCTGAGGCCCAGGGAGCCTCCTGAGTGCATGTCAGGGCAGAGATGAATGGAGAGGGAACTGGATAGGTATGCAAGGCCAGACAGAGAAGAACCTGGTATGTCATGTTTAGCTTCTAAACTAAAACATCTCGAAAACTTCCTAAAATTCCATATGAAATGCAAATGGCGCTGGATTTCAGCTTTTGCTGGTAACCGTGGCAGCTCCCCTACACTGGTTGCTGAGTGAGGGCAGGGCCATCTGCTTTGCTCACCACCCTACCCGAGGCTTGCAACACTGCCTGGCACAGAGCGGGCCTGGGGAGTTTACTCGGGCTGAAATGCCTTCCTTCATTCTTTCTCCTCCGGGCCTCCCATCTGCCTCAGTGAACTGGAAAAAGAGGAAGAGTGCCAGCCATAGCCTTCCATGCTTCCAATGGCCTCTGGAGTTCACGAACAGGTCTTCACCTTTCTGGGCTGCTTCCAAGTGTACACAATGGAGCTCACCACTCCCCACTGCCCAGGACTGCTGTGTAGCTCAAATGAATTCGTGGAAAGTGCCCCCTAAAAATGAAAAGATGCCTCTGGAGACCTTGCGCACACCGAGTGGCACGACCTGCAGAGGTTACCGCCAGGTTTCCATCCTCCGCGCCCGGGAACCGCCTGTCCGGCCAAAGGGTGACTGACTCAGGGCCGGGGCGCCTCCCAGCCCTCCGGCGGAAAGCCACTGCAAAACCGCTTAAGGCGCGTTTGGAAGAGGCGGCCCCGCAGCCCCGAGGGCCTGGGGCCCTGAGTCCCAACTGCCCACCCCCAGTATTCGCAGCGGACCCCACCGGCACCCAGTCCCTGGCACCCCCGCCTCGCGCGGCGGCGGGGAGAAAAGGGTGGAGCGGTCCGACTCCCGCAGCCAATGAAAGCTGCGGGTTCCTGGTCCGATCCCCGGCGCGGCTCGCCATTGGTCGCCGCCCGGGTCTCGGCCCACCGAACCTCGGCGACCCGAGCCAATCGCGAGGCGGCGGGCGATCCCGGGCTCCCCGGGCTGTGGGCTACAGGCGCAGAGCGGGCCAGGCGCGGAGCTGGCGGCAGTGACAGGAGGCGCGAACCCGCAGCGCTTACCGCGCGGCGCCGCACC

**Supplementary data 6. Extended lanes (non-cropped) for Figure panel 3D.**


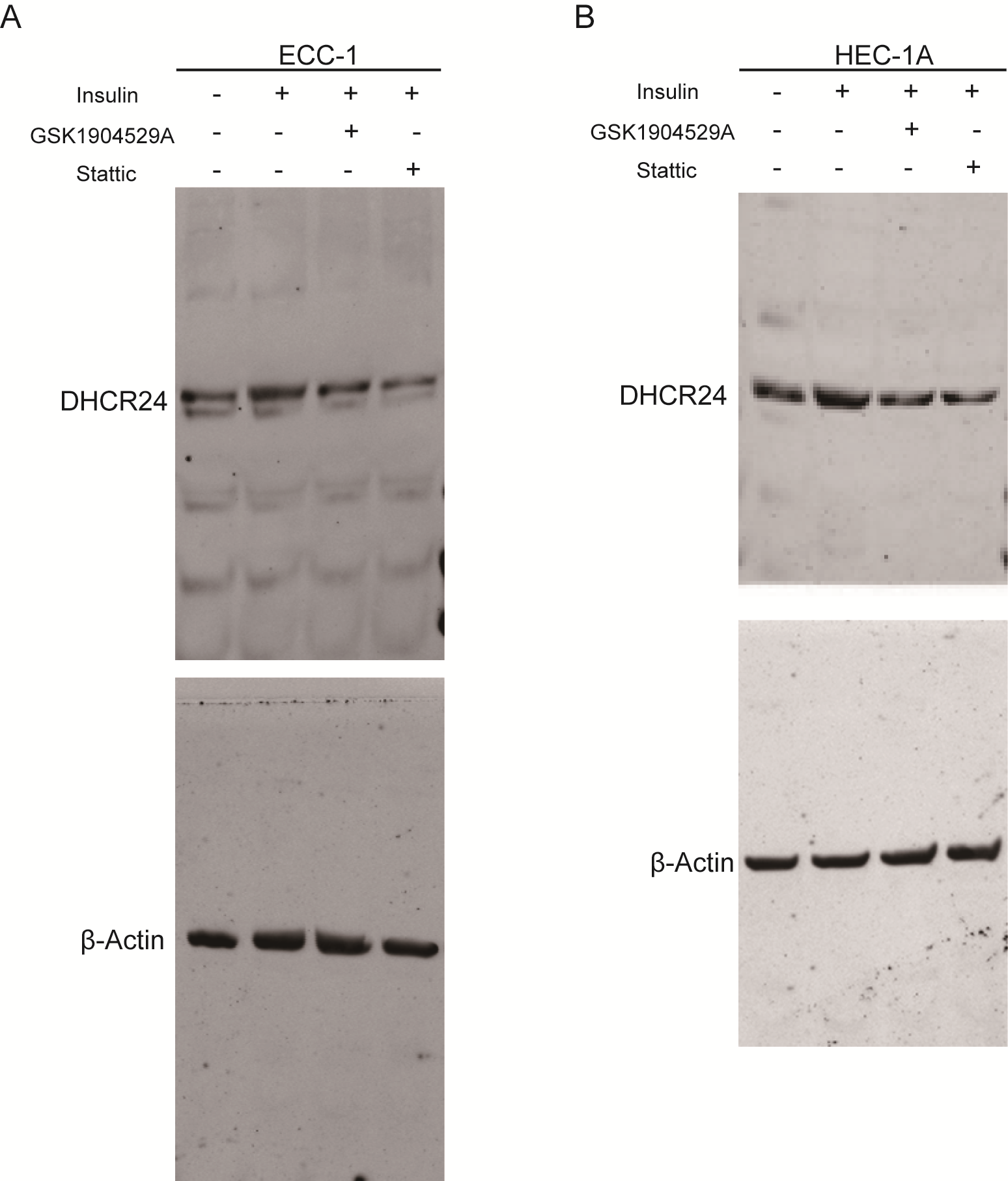


**(A)** Western blots in EEC-1 cell line for figure panel 3D. (**B)** Western blots in HEC-1A cell line for figure panel 3D
